# The Spectrum of Bacille Calmette–Guérin Diseases in Children—A Decade of Data from Neonatal Vaccination Settings

**DOI:** 10.3390/vaccines9020150

**Published:** 2021-02-13

**Authors:** Noora Al Busaidi, Prakash KP, Amina Al-Jardani, Nashat Al-Sukaiti, Salem Al Tamemi, Bader Al-Rawahi, Zaid Al Hinai, Fatma Alyaquobi, Seif Al-Abri, Amal Al-Maani

**Affiliations:** 1 Medical Microbiology Program, Oman Medical Specialty Board, Muscat 132, Oman; noora9225@hotmail.com (N.A.B.); fatmayaquobi@gmail.com (F.A.); 2 Directorate General for Diseases Surveillance and Control, Ministry of Health, Muscat 100, Oman; drprakashkp@gmail.com (P.K.); aksaljardani@gmail.com (A.A.-J.); baderalrawahi4@gmail.com (B.A.-R.); salabri@gmail.com (S.A.-A.); 3 Central Public Health Laboratories, Directorate General for Diseases Surveillance and Control, Ministry of Health, Muscat 100, Oman; 4 Department of Child Health, Royal Hospital, Ministry of Health, Muscat 111, Oman; nashatalsukaiti@yahoo.com; 5 Department of Child Health, Sultan Qaboos University Hospital, Al Khod 123, Oman; tamemi@squ.edu.om (S.A.T.); zaidhinai@gmail.com (Z.A.H.); 6 Department of Infection Prevention and Control, Directorate General for Disease Surveillance and Control, Ministry of Health, P.O. Box 393, Muscat 100, Oman

**Keywords:** BCG, vaccine-related disease, children, abscess, lymphadenitis, immunodeficiency, disseminated disease, Oman

## Abstract

In this paper, we present a multicentre record-based descriptive study used to estimate the incidence and characterize the spectrum of confirmed bacille Calmette–Guérin (BCG) vaccine-related disease among children in Oman. This study included all children (age ≤ 14 years) who had culture and/or polymerase chain reaction (PCR)-confirmed BCG disease from January 2006 to December 2018, as identified from Central Public Health Laboratory data and International Classification of Diseases coding of an electronic patient information system. In total, 88 children confirmed to have BCG disease were included in the study, making an average incidence of 9.2 cases per 100,000 vaccinated neonates. The males comprised 65.9%, Omanis 93.2%, and the median age of presentation was 4 months in children with BCG disease. The most common type of disease was BCG abscesses (72.4%). Children with immunodeficiency and those presenting within 6 months were found to have a more severe and disseminated disease. In total, 28 children had immunodeficiency. The age of presentation and type of BCG disease was significantly associated with immunodeficiency status. The majority of cases required therapy (both medical and surgical) and recovered well. The incidence of laboratory-confirmed BCG vaccine-related disease was low in Oman supporting continuing the use of the BCG vaccination practice at birth.

## 1. Introduction

Bacille Calmette–Guérin (BCG) is a live vaccine derived from a strain of *Mycobacterium bovis* first used by Calmette and Guerin at the Pasteur Institute in Lille, France. It was administered to humans for the first time in 1921 and introduced to the global Expanded Programme on Immunization in 1974 [1]. BCG remains the only vaccine available for the prevention of tuberculosis (TB) disease (73% against meningitis and 77% against miliary TB) with an estimated global coverage of 100 million children per year [2]. The vaccine was introduced in Oman in 1981 and is administered intradermally to all neonates (0.05 mL live attenuated vaccine, *M. bovis*, Tokyo strain) unless contraindicated [3]. 

Although the safety of BCG vaccine for children is well established, vaccine-induced infection has occasionally been reported with an incidence of approximately 1:10,000–1:1,000,000. Disease severity ranges from benign local to disseminated lethal disease, especially in immunocompromised children [4]. The term BCG-related disease is considered when there is the involvement of distant anatomical site(s) beyond the BCG administration site and ipsilateral lymph node(s). The aetiologies of BCG-related diseases can be due to the vaccine itself (e.g., technical errors, vaccine strain), the technique of administration (e.g., faulty intradermal injection), or to the patients’ immunological status, leading to BCG disease [5]. In studies from Iran, Canada, and the United Arab Emirates, the prevalence of adverse reactions was 0.046, 0.93, and 0.6%, respectively [6,7,8]. In Oman, two surveillance studies were published regarding adverse BCG events between 1995 and 2015. The second-decade study found that the BCG vaccine resulted in more adverse events per dose given (37.9/100,000 doses), mostly due to adenitis (82.1%). Since it was passive surveillance data, there may be underreporting of different types of BCG vaccine-related diseases, patient characteristics, and outcomes [9]. 

Recent advanced diagnostic tools, especially molecular tests, helped to distinguish BCG disease from other mycobacterial infections such as TB [5]. The diagnosis of BCG-related disease in children usually triggers investigations for an underlying immunological defect. Unless there is a family history of immunodeficiency disorders, every neonate would receive a routine BCG vaccine after birth, which may lead to BCG-related disease with significant morbidity and mortality in previously undiagnosed children or families [10,11]. The main objective of this study was to estimate the incidence and characterize the spectrum of confirmed BCG vaccine-related disease among children in Oman. This study explores the underlying diseases and outcomes of our cohort with a review of the literature from other parts of the world. 

## 2. Materials and Methods

The study was a multicentre record-based descriptive study of confirmed BCG-related diseases in children (age ≤ 14 years) diagnosed and followed-up during the period from January 2006 to December 2018.

BCG-related disease case definition: A clinical presentation with local, regional, or disseminated infection with proof that it was caused by *M. bovis* BCG from a clinical sample, either by microbiological culture or molecular diagnosis (PCR). The Central Public Health Laboratory, a national reference laboratory for TB and non-tuberculous mycobacteria, provided the line-list of all positive cases. We gathered demographic and clinical details of patients from electronic patient records at two paediatric national referral centres (Royal Hospital and Sultan Qaboos University Hospital). The data collection sheet used included demographic details, clinical details, microbiological and histological investigation, immunological investigations, type of treatment, and the outcome for each patient. A disease that included lymphadenitis, abscesses, osteomyelitis, and disseminated disease was further classified into local, regional, distant, and disseminated based on type and occurrence [5].

### 2.1. Microbiological Studies

In the preliminary period of the study (2006–2015), only conventional methods were available and used to identify and to differentiate *M. bovis* BCG and other members of the *Mycobacterium tuberculosis* complex (MTBC) following culture. The growth on the Löwenstein–Jensen medium of *Mycobacterial* strain resistant to pyrazinamide was indicative of BCG. In 2015, the laboratory started using molecular-based methods to aid in the diagnosis of BCG vaccine-related disease because it is part of mycobacterium complex. GeneXpert (Cepheid, Sunnyvale, CA, USA) and MTBC genotype assay by Hain (HAIN Lifescience, Nehren, Germany) provided positive results from the culture to diagnose BCG vaccine-related disease. Direct detection using MTBC was used only in two cases from the sample to confirm diagnosis because there was no growth. 

### 2.2. Data Analysis

The data was compiled in the Epidata software (Epidata Association, Odense, Denmark) and was analysed using SPSS version 24 (IBM, Armonk, NY, USA). Incidence was calculated using the number of patients who developed BCG-related diseases per 100,000 vaccinated neonates per year. The proportion for each type of disease was calculated from the total occurrence of BCG-related disease. Median, interquartile range (IQR), and median chi-square test was applied for the age variable. Pearson chi-square and Fisher’s exact test statistics were calculated for different group variables. Multiple regression analysis was done for various categories such as gender, age, symptoms, type of treatment, and outcome on immunodeficiency. *p* < 0.05 was considered statistically significant.

## 3. Results

**Figure 1:**
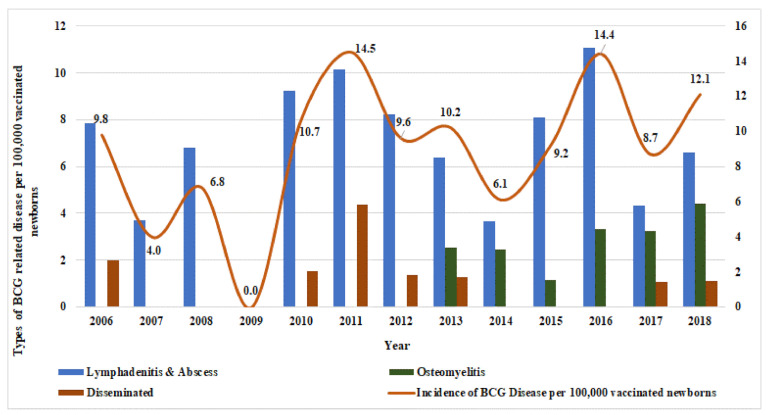
Incidence of total bacille Calmette–Guérin (BCG) vaccine-related disease and each type per 100,000 vaccinated neonates.

During the 13-year study period, there were 88 laboratory-confirmed cases of BCG vaccine-related disease, which accounted for an average incidence of 9.2 cases per 100,000 (0.009%) vaccinated neonates. The incidence rate varied from 0.0 to 14.5 cases per 100,000 vaccinated neonates. Figure 1 depicts the year-wise incidence rates for the total and type of BCG vaccine-related disease per 100,000 vaccinated neonates. It is noted in the figure that osteomyelitis cases have been reported since 2013 but disseminated cases have been reported since 2006.

In 84 out of 88 cases, we had complete information in the medical electronic system to show descriptive statistics such as gender, nationality, and sign/symptom (Table 1). The majority of the study subjects were male (65.9%) and Omani (93.2%). The predominance of the male gender was not shown to be statistically significant in association with different types of BCG diseases. Those who presented at an age ≤6 months were found to have more severe and disseminated disease (test statistic: 8.43, *p*-value: 0.03). Moreover, those who had a fever were found to have more of an association with distant and disseminated disease (test statistic: 16.81, *p*-value: <0.005). The disease occurred on the left side of the body in 76.5% of cases, occurred on other sites (Trunk, right upper, or lower limbs) in 12.8% of cases, and was generalized in 10.7% of cases. The left side was likely more common because vaccine injections are given in the left shoulder. 

Of all 88 cases, the most common type of BCG vaccine-related disease was lymphadenitis with or without abscesses (72.7%), followed by osteomyelitis (17.1%) and disseminated disease (10.2%). The diagnosis was made mainly by a positive culture in 94% of cases. Five cases had negative culture but three of them had positive GeneXpert; the other two had positive PCR by Hain directly from the tissue/pus sample. Acid-fast bacillus (AFB) was found to be positive in 70.5% of cases. Although GeneXpert started in late 2015 and was used for only 26 samples, results were positive except in two cases that had a positive culture. Similarly, for PCR, which was used from growth, and started in late 2016 and used in 21 samples. Results were positive in all cases except for one, which had a positive result from GeneXpert. Out of the 88 diagnosed cases, 41 had histopathological results available and the granulomatous changes in 35 of them supported the diagnoses of BCG vaccine-related disease.

The majority of cases required medical and surgical treatment in all types of BCG vaccine-related disease. All cases of osteomyelitis and disseminated diseases were treated with anti-TB medications. There was no statistically significant relation between diagnosis, treatment, and outcome for each type of BCG-related disease (Table 2). There were no reported adverse medical or surgical treatment events in 75% of cases. Moreover, 8% of cases had drug-related transaminitis, 4.5% had wound infection, 2% had drug-related neutropenia, and 4.5% had other adverse events, including allergy and acute kidney injury.

There was no immunological workup done in 54.5% of the 88 cases. In total, 28 cases were labelled as immunodeficiency, 12 of which were diagnosed with a primary immunodeficiency disease and 16 remained by the study’s end with undefined immunodeficiency diagnosis. Among lymphadenitis and abscess categories, there were four cases of undefined immunodeficiency, two cases of chronic granulomatous disease (CGD), one case of severe combined immunodeficiency (SCID), one case of hemophagocytic lymphohistiocytosis, and one case of myeloid differentiation primary response 88 (MyD88). Among the osteomyelitis category, all 10 cases had undefined immunodeficiency. Among the disseminated category, there were four cases of SCID, two cases of interferon-gamma deficiency, one case of CGD, and two cases of undefined immunodeficiency. There was a statistically significant difference between disease outcome and immunodeficiency status in each type of BCG-related disease (Table 3).

The median age at diagnosis of immunodeficiency was seven months (IQR: 5–11.5). The median age of clinical symptom presentation of BCG vaccine-related disease was four months (IQR: 2–9). The age of presentation of clinical symptoms was statistically significant (median chi-square value: 11.49, df: 2, *p*-value: 0.003) on immunodeficiency status (immuno-deficient, immunocompetent, and reference category was no immunological workup). Children with immunodeficiency are more likely to have BCG vaccine-related disease presentation early (≤6 months) compared to others. 

Most affected children had recovered well (60%): three cases of osteomyelitis had a deformity and five died. All of the children who died were diagnosed with primary immunodeficiency (four with SCID, and one with undefined immunodeficiency) and the direct cause of death was related to other complications of immunodeficiency, not the BCG disease.

Multiple regression analysis for the variables of gender, age, symptoms of presentation, type of treatment, and the outcome did not show statistical significance in predicting the immunodeficiency status of the child. However, the type of BCG vaccine-related disease (lymphadenitis/abscess, osteomyelitis, or disseminated) was a statistically significant (odds ratio (OR): 0.42, 95% CI: 0.79–2.25, *p*-value: 0.04) predictor of the immunodeficiency status (immuno-deficient, immunocompetent, and reference category had no immunological workup). This statistical significance indicates that children with an immunodeficiency are more likely to develop a severe disease, such as osteomyelitis and disseminated disease compared to others, thus stressing the importance of immunological workup for such cases.

## 4. Discussion

Studies of BCG-related disease among children worldwide, especially from low- and middle-income countries, are rare. This study from Oman where BCG is given at birth to neonates with an almost 100% coverage rate has shown that the average incidence of laboratory-confirmed BCG vaccine-related disease is 9.2 cases per 100,000 vaccinated neonates (0.009%). This is a very low incidence compared with the vaccines’ adverse effects reported in previously published studies as it has been limited only to the moderate to severe forms that would require taking laboratory samples for confirmation and treatment [6,7,8,9].

In general, BCG received within one month of birth is the most important risk factor for developing a reaction followed by the method of application, the number of viable bacilli, and the type of strain used in immunization [12,13]. Thus, globally, the incidence of complications in the pre-HIV era has varied and ranged from 10 to 500 per 100,000 vaccinated children [14,15]. In our study, incidence varied from 0 to 15 per 100,000 vaccinated neonates and the country is implementing an antenatal HIV screening program which makes our setting more comparable to the global pre-HIV era. This variation can be attributed to the changes in the practice of administering vaccines with periodic training of staff and/or improvement in diagnostics, especially after the introduction of GeneXpert. However, the vaccine strain used in Oman and at-birth timing for giving the vaccine remained the same throughout. 

BCG vaccine-related diseases are classified as local, regional, distant, and disseminated disease [5]. In our study, there was no BCG-related disease reported in HIV-infected children, perhaps due to the implementation of antenatal screening programmes for the retroviral infection that have operated since 2009 and the low incidence of paediatric HIV in the country [16]. Similar to our study, lymphadenitis has been the most commonly reported BCG-related disease in many studies that included clinical and laboratory diagnosed cases [17,18]. However, the Federal University of São Paulo study showed the most common disease was abscess at the BCG injection site or lymph node [19]. 

The median age of presentation in our study was four months, which was in concordance with other reports in the literature [17]. The most common site was the axillary, nearest to the BCG site, which was also reported in other studies [7,8]. 

Disseminated infection and osteitis in immunocompetent children are extremely rare and its incidence is reported to range from 0.19 to 1.56 per million doses [14]. However, a Canadian study showed a much higher incidence than previously reported at 205 cases per million doses [20]. The severe disease risk is high in HIV-infected children and is considered a significant public health issue [21,22]. Thus, HIV-infected children, even if asymptomatic, are not vaccinated until confirmed negative, per the revised World Health Organization vaccination guidelines [23]. In our study, we looked into the spectrum of severity of cases and as expected, the mild and moderate cases managed clinically and recovered well. Conversely, disseminated manifestations were restricted to patients with primary immunodeficiency and we had no HIV-infected children or children who suffered acquired immune deficiencies.

Nearly 32% of patients in our study were suspected to have underlying primary immunodeficiencies, but only 15%were confirmed to have specific primary immunodeficiency (PID) conditions, which were mostly due to limitations in the diagnostic tests for children presenting with BCG osteomyelitis where routine workup was not productive. The most common PID was SCID followed by CGD. A previous local study of 90 PID cases found that 61% were male; 12% had BCG disease. The estimated prevalence of PID in Oman is 4.5 cases per 100,000, and the median diagnosis age is 24 months [24]. Although it was not statistically significant, the predominance of the male gender in our study was thought to be because of the predominance of PID conditions, yet this was rejected based on the result of the multiple regression analysis. Similar complications were seen in countries with similar incidence and prevalence of primary immunodeficiency, where the BCG vaccine is universal [1,2,3,4].

Similar to our study, children with a primary immunodeficiency suffered from a disseminated or more severe disease in Iran (10/17, 59%) and China (43.2%) [25,26]. However, in Pakistan, none of the children with immunodeficiency developed severe diseases [27]. BCG vaccine-induced disease, whether local, regional, or disseminated, can lead to significant parental anxiety, increased infant morbidity, and even death but there is a lack of clear evidence-based treatment protocol for this group of diseases, especially in the immunocompromised host [6]. The best approach to decrease risk of BCG-related diseases in immunocompromised children would be to screen for family history of such conditions and avoid BCG vaccination at birth for this high-risk group until the child has proven otherwise healthy. 

Medical therapies include anti-tuberculosis drugs and surgical options meant to lessen the risk of complications, such as suppuration, but their efficacy has not yet been adequately evaluated [28,29]. A systematic review found no evidence of benefit in using oral antibiotics (e.g., isoniazid, erythromycin, or a combination of isoniazid plus rifampicin) to treat local or regional BCG-induced disease [4]. Similar to other studies, anti-TB drugs were used in our study in 44% of cases [18]. The duration of anti-TB medication depends on the type of complication. Those who suffered from osteomyelitis required a longer duration compared to disseminated disease because the later patients died during suppressive therapy before the completion of anti-TB drugs. Surgical excision is not recommended as a first-line approach but may be needed after an aspiration failure [4]. The majority of our cases that required surgical intervention were managed by incision and drainage. 

It is clear from this study and a review of the literature that BCG-related disease remains of significant morbidity and mortality in a small group of immunodeficient children with no clear management guidelines. Some countries, especially those with low TB incidence, have delayed BCG administration until 3 and 6 months to avoid BCG complications in infants born with immunodeficiency, and others went to selective vaccination for high-risk infants instead of taking a universal approach [10,11,30]. The experience from Greenland, Tunisia, and Bahrain of increased incidence and/or severity of TB especially in children after waving out universal neonatal BCG vaccine is a call for careful consideration before adopting such an approach even by low incident countries [30,31,32]. The rate of all TB forms in Oman decreased from 21.4 per 100,000 population in 1991 to 5.9 per 100,000 population in 2018, remaining remained constant since then. Non-national migrant populations from high endemic countries accounted for 60% of the annual TB cases in Oman [33]. High vaccination coverage and the directly observed treatment, short course (DOTS) program have largely contributed to the decrease in TB cases in Oman. With a low incidence of severe complications from the BCG, it will be recommended to continue at-birth vaccination in the process of a national TB elimination strategy. 

The strengths of this study were that the data was taken from multicentre national data and included the laboratory-confirmed BCG-related disease, which indirectly reflected the moderate to severe complications of the vaccine and outcomes that included the uncovering of immunodeficiency status. The main limitation of our laboratory study was missing clinically diagnosed cases and cases diagnosed abroad. Being a record-based study, incomplete and inaccurate data, clinical details, and immunodeficiency workup was noticed in some cases.

## 5. Conclusions

The incidence of laboratory-confirmed BCG vaccine-related disease was low in Oman. The disease severity was related to age at presentation and the child’s immunodeficiency status. Although most cases required both medical and surgical treatment, they ultimately recovered well. BCG vaccination is overall safe and should be considered in the process of TB elimination. 

## Figures and Tables

**Table 1:** Descriptive statistics of BCG vaccine-related disease cases according to gender, nationality, signs, and symptoms.

Variable	BCG Vaccine-Related Disease Cases (*N* = 84)				Fisher’s Exact Test Value, (*p*-Value) *
	Local (9, 10.7%)	Regional (54, 64.2%)	Distant (12, 14.2%)	Disseminated (9, 10.7%)	
**Age (months)**					
≤6	7	37	3	6	8.43, (0.03)
>6	2	17	9	3	
**Gender**					
Male	6	40	7	4	3.85, (0.27)
Female	3	14	5	5	
**Nationality**					
Omani	7	50	12	9	3.33, (0.28)
Non-Omani	2	4	0	0	
**Signs and symptoms ^#^**					
Fever and inflammatory signs/symptoms	1	13	6	8	16.81, (<0.005)
Only inflammatory signs/symptoms	8	41	6	1	

* Fisher’s exact test, *p* < 0.05 considered statistically significant; ^#^ Inflammatory signs/symptoms: one or multiple sign/symptoms of pain/swelling/erythema/discharge.

**Table 2:** Diagnosis, treatment, and outcomes for each type of BCG vaccine-related disease.

Variable		BCG Vaccine-Related Disease Cases			Pearson’s Chi-Square (*p*-Value)
		Lymphadenitis and/or Abscess (*N* = 64)	Osteomyelitis (*N* = 15)	Disseminated (*N* = 9)	
**Diagnosis**					
Acid Fast Bacillus stain (AFB) **(*N* = 87)**	Positive *N* (% from Column)	47 (73%)	7 (47%)	8 (89%)	4.6, (0.10)
	Negative *N* (% from Column)	17 (27%)	7 (47%)	1 (11%)	
Culture **(*N* = 88)**	Positive *N* (% from Column)	62 (97%)	13 (87%)	8 (89%)	2.9, (0.23)
	Negative *N* (% from Column)	2 (3%)	2 (13%)	1 (11%)	
Molecular * **(*N* = 37)**	Positive *N* (% from Column)	24 (38%)	11 (73%)	2 (22%)	-
	Negative *N* (% from Column)	0	0	0	
**Type of treatment (*N* = 88)**					
Medical ** only *N* (% from Column)		2 (3%)	2 (13%)	2 (22%)	-
Surgical only *N* (% from Column)		7 (11%)	0	0	
Medical and surgical *N* (% from Column)		48 (75%)	13 (87%)	7 (78%)	
No treatment information *N* (% from Column)		7(11%)	0	0	
**Use of anti-TB (*N* = 84)**					
Yes *N* (% from Column)		13 (28%)	15 (100%)	9 (100%)	
No *N* (% from Column)		47 (73%)	0	0	

* Molecular diagnosis—PCR/GeneXpert. ** Medical treatment—includes the use of anti-TB drugs and other antibiotics.

**Table 3:** Outcome and immunodeficiency status in each type of BCG vaccine-related disease.

Variable	BCG Vaccine-Related Disease Cases (*N* = 88)			Fisher’s Exact Test Value, (*p*-Value) *
	Lymphadenitis and/or Abscess (*N* = 64)	Osteomyelitis (*N* = 15)	Disseminated (*N* = 9)	
**Outcome *N* (% from total)**				
Recovered	38 (43%)	11 (13%)	4 (5%)	27.98, (<0.005)
Recovered with deformity	0	3 (3%)	0	
Death	1 (1%)	0	4 (5%)	
No data available	25 (28%)	1 (1%)	1 (1%)	
**Immunodeficiency**				
Immunodeficiency	9 (10%)	10 (11%)	9 (10%)	43.03, (<0.005)
No immunodeficiency	8 (9%)	4 (5%)	0	
No workup	47 (53%)	1 (1%)	0	

* *p* < 0.05 considered statistically significant.

## Data Availability

The data of this research is part of residency program research project of the first author and is not available on open resource but can be provided based on request from the correspondence author (project supervisor).
